# Characterization of healing following atherosclerotic carotid plaque rupture in acutely symptomatic patients: an exploratory study using in vivo cardiovascular magnetic resonance

**DOI:** 10.1186/1532-429X-13-64

**Published:** 2011-10-27

**Authors:** Zhongzhao Teng, Andrew J Degnan, Umar Sadat, Fang Wang, Victoria E Young, Martin J Graves, Shengyong Chen, Jonathan H Gillard

**Affiliations:** 1 University Department of Radiology, University of Cambridge, UK; 2 Department of Engineering, University of Cambridge, UK; 3 Department of Surgery, Cambridge University Hospitals NHS Foundation Trust, Cambridge, UK; 4 College of Computer Science and Technology, Zhejiang University of Technology, Hangzhou, China

**Keywords:** carotid atherosclerosis, rupture, healing, curvature, roughness, CMR

## Abstract

**Background:**

Carotid plaque rupture, characterized by ruptured fibrous cap (FC), is associated with subsequent cerebrovascular events. However, ruptured FC may heal following stroke and convey decreased risk of future events. This study aims to characterize the healing process of ruptured FC by assessing the lumen conditions, quantified by the lumen curvature and roughness, using in vivo carotid cardiovascular magnetic resonance (CMR).

**Methods:**

Patients suffering from transient ischemic attack underwent high resolution carotid MR imaging within 72 hours of the acute cerebrovascular ischemic event. CMR imaging was repeated at 3 and 12 months in 26 patients, in whom FC rupture/erosion was observed on baseline images and subsequent cerebrovascular events were recorded during the follow-up period. Lumen curvature and roughness were quantified from carotid CMR images and changes in these values were monitored on follow-up imaging.

**Results:**

Healing of ruptured plaque was observed in patients (23 out of 26) without any ischemic symptom recurrence as shown by the lumen surface becoming smoother during the follow-up period, characterized by decreasing maximum lumen curvature (p < 0.05), increasing minimum lumen curvature (p < 0.05) and decreasing lumen roughness (p < 0.05) during the one year follow-up period.

**Conclusions:**

Carotid plaque healing can be assessed by quantification of the lumen curvature and roughness and the incidence of recurrent cerebrovascular events may be high in plaques that do not heal with time. The assessment of plaque healing may facilitate risk stratification of recent stroke patients on the basis of CMR results.

## 1. Background

Recent advances in high resolution cardiovascular magnetic resonance (CMR) of the carotid artery allow for the identification of plaque components only before seen on histology following carotid endarterectomy [1]. One component, the fibrous cap, acts a protective structure enclosing the lipid-rich atheroma; in vulnerable plaques, matrix metalloproteinases produced by inflammatory cells can degrade the fibrous cap, exposing plaque material to the lumen, and thereby propagating a thromboembolic cascade capable of producing distal cerebrovascular occlusion and ischaemia [2]. Fibrous cap (FC) rupture, therefore, could serve as an imaging indicator of vulnerable plaque. This finding has been associated with subsequent cerebrovascular events (hazard ratio (HR): 7.39 in symptomatic patients [3] and HR:17.0 in asymptomatic patients [4]). Angiographic evidence of plaque ulceration increases the risk of stroke in symptomatic patients with high grade (≥ 70%) stenosis [5] and asymptomatic patients [4]. Being a dynamic structure with the ability to heal under favourable circumstances, carotid atheroma may heal following rupture or erosion [6,7]. However, the exact mechanism by which this healing process occurs and alters the lumen condition has not been examined to date. In this study we explore the changes that may occur to the ruptured FC over time by characterizing the luminal contour changes, quantified by parameters such as lumen curvature and lumen roughness.

## 2. Methods

Patients were recruited for this study after having experienced a cerebrovascular event. The baseline (0 M) images were acquired within 72 hours of the onset of ischemic cerebrovascular symptoms in a 1.5 Tesla magnetic resonance (MR) system (Signa HDx GE Healthcare, Waukesha, WI) with a 4-channel phased-array neck coil (PACC, Machnet BV, Elde, The Netherlands). Twenty-six patients with image-delineated FC rupture, ulceration or erosion were imaged again at 3 months (3 M) and 12 months (12 M). Following the event, it was ensured that patients were on the best medical therapy i.e. anti-platelets, cholesterol lowering medication and antihypertensive medication (if required). The protocol was reviewed and approved by the regional research ethics committee and all patients gave written, informed consent. The criteria for inclusion in the study were: (1) internal carotid artery stenosis of 30-69% on duplex imaging during screening assessment; (2) sufficient MR image quality to identify the lumen wall and outer boundary of the arterial wall; (3) image quality (IQ) > 3 were included for morphological analysis (The image quality (IQ) was rated before review by using a previously published five-point scale [8,9]); and (4) normal heart rhythm, confirmed by 24-hour Holter monitoring and normal transthoracic echocardiography in patients where a cause of stroke other than carotid artery disease was suspected. Exclusion criteria included: (1) previous endarterectomy of the symptomatic carotid artery; (2) cardiac arrhythmias; (3) known coagulation/clotting disorder potentially responsible for patient's symptoms; (4) patients undergoing thrombolysis following the acute cerebrovascular event; and (5) clinical contraindications to CMR such as inner ear implants, metallic implants and cardiac pacemakers.

### CMR Image Acquisition and Analysis

During the CMR scan, movement artefact was minimized using a dedicated vacuum-based head restraint system (VAC-LOK Cushion, Oncology Systems Limited, UK) to fix the head and neck in a comfortable position and allow close apposition of the surface coils. After an initial coronal localizer sequence, axial 2D time-of-flight (TOF) MR angiography was performed to identify the location of the carotid bifurcation and the region of maximum stenosis on each side. Axial images (of 3 mm thickness) were acquired to ensure that the entire plaque on the previously symptomatic side was imaged. Approximately, 10 to 12 high resolution MR slices were acquired for each plaque. The following electrocardiography (ECG)-gated fast spin echo pulse sequences were used: T_1_-weighted (repetition time/echo time: 1 × RR/7.8 ms) with fat saturation, Proton Density (PD)-weighted (repetition time/echo time: 2 × RR/7.8 ms) with fat saturation, T_2_-weighted (repetition time/echo time: 2 × RR/85 ms) with fat saturation; and short tau inversion recovery (STIR) (repetition time/echo time/inversion time: 2 × RR/42 ms/150 ms). The field of view was 10 × 10 cm^2 ^and matrix size 256 × 256. The in-plane spatial resolution achieved was 0.39 × 0.39 mm^2^. This MR protocol has been used previously by our group for carotid plaque imaging [10].

**Figure 1:**
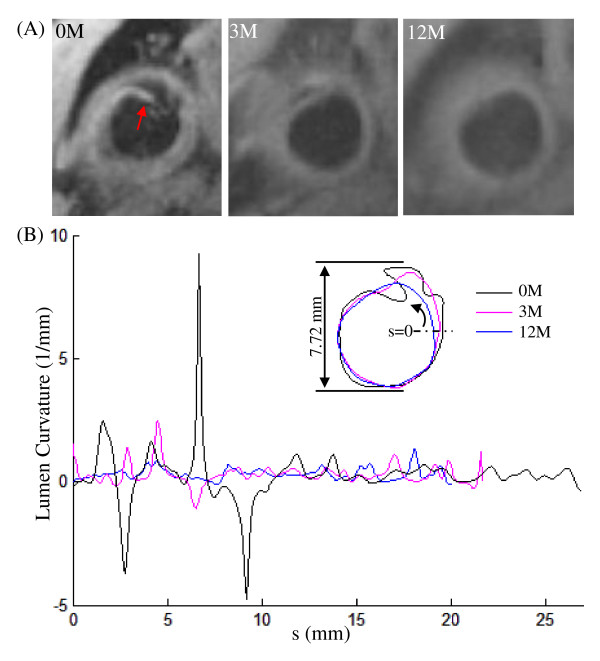
**In vivo CMR-images showing the healing process which can be characterized using lumen curvature**. (A): T_1 _CMR image at baseline (0 month; 0 M) showing ruptured fibrous cap (red arrow) and the healing observed after 3 and 12 months (3 M and 12 M); (B): the corresponding lumen contours and the lumen curvature curves.

The researcher responsible for the image segmentation was blinded to the patient symptom status and follow-up information. The analysis of lumen curvature and roughness was performed by a separate researcher, who was not involved in the image segmentation. The lumen condition was evaluated using the images from sequences of T_1_, PD, T_2_, STIR and TOF, and the corresponding contour was manually drawn in CMR Tools (London, UK) (Figure 1A). Fibrous cap rupture was defined as complete disruption of the low signal band on T_1 _images and high signal band on STIR images and fibrous cap erosion differed in only incomplete disruption of the FC signal using previously described morphologic and contrast patterns on multispectral imaging [11,12]. The MR slices with different imaging sequence were matched according to the slice position, and slices at different scan times were aligned with reference to the carotid bifurcation and considering the 3-mm slice interval in order to ensure slice matching. Moreover, only confirmed slice matches were used in comparing measurements across time points. The analysis was performed with slices presenting FC rupture/erosion at baseline image and others were excluded.

### Definition of Lumen Curvature and Roughness

**Figure 2:**
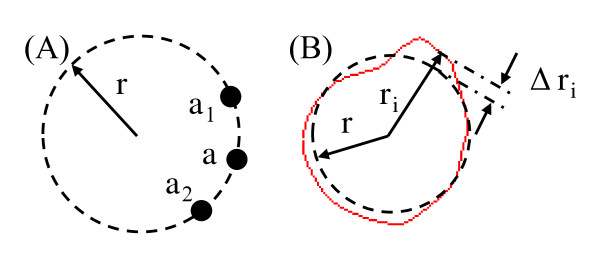
**Schematic drawing showing the definition of lumen curvature and roughness**. (A): The lumen curvature at *a *can be computed from the circle determined by *a *and the two adjacent points, *a*_1 _and *a*_2_; (B): The definition of roughness. Red line: lumen contour; Dash black line: the circle best fitting the lumen contour.

Lumen curvature has been recently introduced to describe the vulnerable site (plaque shoulder), where the rupture likely occurs [13]. Briefly, as shown in Figure 2A, the curvature at point *a *is the reciprocal of the radius (1/*r*) of the circle determined by *a *and the two adjacent points, *a*_1 _and *a*_2_. FC rupture, ulceration and erosion will all lead to an irregular lumen surface. It can be captured by the sharp peaks of the curvature curve along the lumen length. As shown in Figure 1A, at the onset of ischemic symptoms (0 months), FC rupture and erosion were observed. The curvature curve reached its positive peaks at the crack positions (marked by green and brown arrows in Figure 1) and negative peaks at the positions in which tissue encroached upon the lumen (red arrow in Figure 1; black line in Figure 1B).

Extreme values (maximum and minimum) of curvature represent the local lumen irregularity and they were used to characterize the local lumen surface change during the imaging follow-up. The surface roughness, initially created to quantify the degree of evenness of a metal surface [14], was introduced to describe the lumen surface of the plaque. It was computed as

$$Roughness=\frac{1}{r}\sqrt{\frac{1}{L}\sum {\left(r_{i}-r\right)}^{2}\text{Δ}l}$$

in which *r *is the radius of the circle best fitting the lumen contour; *r_i _*is the distance between the centre of the circle and the *i*^th ^point on the lumen contour (Figure 2B); *L *stands for the total length of the lumen contour and Δ*l *is the half of the length of the curve connecting *i*-1^th^, *i *^th ^and *i*+1^th ^points. Smaller value of roughness indicates more round lumen shape, that is, a perfect round lumen shape will have roughness being zero.

### Statistical Analysis

For the data of either group not passing the normality test (Shapiro-Wilk test), two-tailed Wilcoxon matched-pairs signed-ranks test was used for the statistical analysis; otherwise, two-tailed student paired t test was used. The statistical analysis was performed in Instat3.06 (GraphPad Software Inc.). A significant difference was assumed if p value < 0.05.

## 3. Results

The demographics of 23 patients with FC defects and without recurrent events during the follow-up period are shown in Table 1. Recurrent events were observed in three of 26 patients within the one year imaging follow-up period. Due to the limited number of patients with recurrence, the analysis focused predominantly on those (n = 23) without any recurrence of ischemic symptoms. In total, 54 CMR slices with lumen defects were traced, in which rupture was observed in 16 (29.6%), erosion in 26 (48.1%) and both of them were observed in 12 (22.2%).

**Table 1:** Patient demographics (n = 23)

	Total number of patients/value
Sex (male; %)	14 (60.9)
Age (years ± SD)	70.1 ± 11.4
Systolic blood pressure (mmHg ± SD)	138.8 ± 20.1
Diastolic blood pressure (mmHg ± SD)	78.4 ± 14.2
Heart rate (beats/minute)	71.1 ± 9.6
Hypertension, n (%)	8 (34.8)
Diabetes, n (%)	1 (4.3)
Renal Impairment, n (%)	2 (8.7)
Ischemic heart disease, n (%)	5 (21.7)
Peripheral vascular disease, n (%)	1 (4.3)
Coronary artery disease, n (%)	1 (4.3)
Previous TIA/Stroke, n(%)	7 (30.4)
Statin used before recruitment, n (%)	16 (69.6)
Aspirin used before recruitment, n (%)	8 (34.8)
ECST defined luminal stenosis (% ± SD)	54.4 ± 15.4
Clinic follow-up period (Days ± SD)	546 ± 186

**Figure 3:**
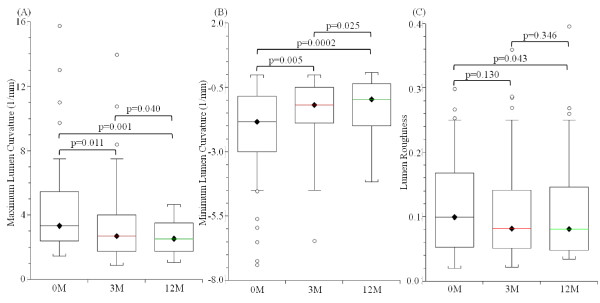
**Comparison of maximum curvature, minimum curvature and lumen roughness over time**. In summary, the lumen became smoother while healing occurred in the patient group (n = 23) without recurrent events. (A): maximum lumen curvature decreased with time; (B) minimum lumen curvature increased with time; and (C) the lumen roughness decreased.

As shown in Figure 1A, healing was observed during the recovery period with the lumen contour becoming smoother at 3 months and 12 months. Corresponding quantitative comparisons between the curvature at baseline, 3 months and 12 months are made in Figure 1B. It can be seen that curvature became much flatter at 12 months. Comparisons of maximum and minimum lumen curvatures of the patient group without any recurrent event are visualized in Figure 3A &3B. The maximum lumen curvature decreased significantly from 3.33 [2.42, 5.43] (median [inter quartile range]; mm^-1^) at baseline (0 M) to 2.68 [1.78, 3.97] at 3 months and 2.53 [1.74, 3.45] at 12 months (p values are 0.011 and 0.001, respectively). The minimum lumen curvature increased from -1.83 [-2.89, -0.92] at baseline (0 M) to -1.19 [-1.86, -0.51] at 3 months and -0.96 [-1.93, -0.36] at 12 months (p values are 0.005 and 0.0002, respectively). Although the roughness decreased from 0.100 [0.054, 0.164] at baseline to 0.082 [0.051, 0.138] at 3 months (Figure 3C), there was no significant difference (p = 0.130). With a further decrease, a significant difference was found at 12 months (0.079 [0.050, 0.130]; p = 0.043).

**Figure 4:**
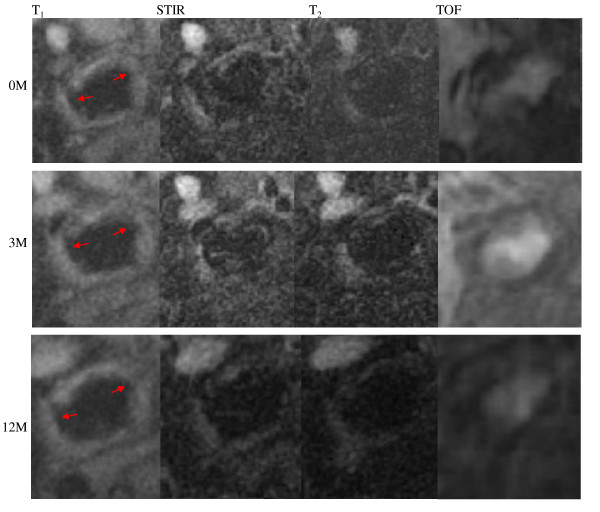
**A patient who suffered from a recurrent event during CMR follow-up period with insufficient healed lumen erosions**.

Although the number of patients with recurrent events was small (n = 3), the evolution of curvature and roughness might be helpful in understanding the healing process. Figure 4 shows the CMR-traced lumen erosion (marked with red arrows) at 0 M, 3 M and 12 M of a patient who suffered a recurrent event. It can be seen that the recovery in this case is insufficient as the lumen defect remained throughout the imaging follow-up period. In total, eight CMR slices with lumen defect were traced for those with recurrence of ischemic symptoms. As listed in Table 2, although after three months, the absolute values of maximum and minimum lumen curvature decreased significantly (p values are 0.025 and 0.007, respectively), the lumen condition worsened at 12 months resulting in no significant differences between baseline and 12 months (p values are 0.625 and 0.872, respectively). There is no improvement in terms of lumen roughness (baseline vs. 3 months, p = 0.742; 0 M vs. 12 M, p = 0.296).

**Table 2:** Changes in lumen curvature in those patients (n = 3) with recurrent events

	0 M	3 M	12 M
Maximum Lumen Curvature (1/mm)	3.06 [2.50, 3.84]	2.30 [1.54, 3.28]	2.91 [2.40, 3.14]
Minimum Lumen Curvature (1/mm)	-2.92 [-4.45, 2.15]	-1.08 [-1.34, -0.94]	-1.82 [-2.02, -1.49]
Roughness	0.12 [0.11, 0.15]	0.12 [0.92, 0.19]	0.17 [0.6, 0.18]

## 4. Discussion

To our best knowledge, this is the first study tracing the healing process after FC rupture, ulceration or erosion, except for a single case report [6] and one recent short report [7], and the only study to use the lumen curvature and roughness to describe this process. The results indicate that healing occurs after the acute ischaemic event in those without recurrence (Figure 1A). In the patient group without any recurrent events, the absolute values of maximum and minimum lumen curvature became smaller during the follow-up period (Figure 3A &3B) as well as the lumen contour roughness (Figure 3C). Such a tendency was less clear in the patient group with subsequent cerebrovascular events. Therefore, it is plausible to hypothesize that plaque is more vulnerable if insufficient healing occurs.

Since, under physiological conditions, plaque is subjected to mechanical loadings from blood flow and pressure, the critical mechanical conditions need to be considered. It is well known that an uneven lumen surface will induce eddy flow or even turbulence, and therefore oscillating shear stress, which will further affect the biomechanical stability of the endothelium [15,16], thereby contributing to plaque progression and eventually FC rupture. On the other hand, high stress concentration within the plaque structure will appear at the site with the largest lumen curvature when the lumen is irregular [17], and the stress magnitude increases with the increasing curvature [18]. Moreover, in both coronary [19] and carotid plaque [20,21] the rupture site is usually associated with a high concentration of stress. Such high stress concentration may dysregulate cytoskeletal gene expression, such as filamin A [22], affecting the cell attachment, and local cell apoptosis [23-25], and therefore impede healing by preventing the formation of new fibrous cap and endothelium.

It has been discovered that healing is a long process and carotid ulcerations persist for a long time [7]. This study confirmed this conclusion. Although healing process occurred over time, most FC rupture/erosion remained after one year and only 5 out of 54 (9.3%) showed healed by visual assessment.

It is important to note that these findings may be confounded by the inherent limitations of current imaging methods. Inadequate spatial resolution (in-plane resolution in this study was 0.39 × 0.39 mm^2^) could mean that roughness in delineation of the lumen contour potentially reflects differences in noise within images. And FC rupture/erosion might be missed due to the 3-mm slice interval. Moreover, other factors such as partial volume effects and slice-matching differences could limit repeatability and need to be addressed systematically in future analyses. Nevertheless, tracing the lumen contour changes in patients with recent symptoms may provide an indicator of the relative risk of future cerebrovascular ischaemia by categorizing patients on the basis of the adequacy of FC rupture healing.

## 5. Conclusions

In conclusion, as a dynamic structure with endothelial repair mechanisms, the fibrous cap has the capability to heal following rupture seen in the setting of acute cerebral ischaemia. Studying the healing process is helpful in understanding how atherosclerotic plaque is stabilized or destabilized and will therefore identify those plaques with the greatest risk of future cerebrovascular events.

## Competing interests

The authors declare that they have no competing interests.

## Authors' contributions

ZT designed the study, processed the data and wrote the manuscript; AD processed the data and revised the manuscript; US recruited patients and revised the manuscript; FW processed the data; VEY recruited patients and processed the data; MJG developed MR sequences; SY processed the data and revised the manuscript and JHG designed the study and revised the manuscript. All authors read and proved the manuscript.

## Source of funding

This research is partly supported by ARTreat European Union FP7 and the National Institute of Health Research, Cambridge Biomedical Research Centre grant.

## Disclosures

None
